# Comparative Effectiveness and Safety of Direct Oral Anticoagulants Versus Vitamin K Antagonists in Elderly Patients With Atrial Fibrillation: A Systematic Review

**DOI:** 10.7759/cureus.85615

**Published:** 2025-06-09

**Authors:** Hafsa Aman, Ayesha Sikandar, Faiza Khawar Dar, Zayam Shahid, Sharen Shibu, Muhammad Usman, Noor Abbas

**Affiliations:** 1 Internal Medicine, Fairfield General Hospital, Bury, GBR; 2 Internal Medicine, Royal Blackburn Teaching Hospital, Blackburn, GBR; 3 Internal Medicine, Dow University of Health Sciences, Civil Hospital Karachi, Karachi, PAK; 4 General Practice, Railway General Hospital, Rawalpindi, PAK; 5 Internal Medicine, Vinnytsia National Pirogov Medical University, Vinnytsia, UKR; 6 Internal Medicine, Jinnah Hospital, Lahore, PAK; 7 Internal Medicine, Services Hospital Lahore, Lahore, PAK

**Keywords:** anticoagulation, atrial fibrillation, bleeding risk, cognitive outcomes, direct oral anticoagulants, doacs vs vkas, elderly, randomized controlled trials, stroke prevention, warfarin

## Abstract

Anticoagulant therapy plays a pivotal role in preventing stroke and thromboembolic complications in elderly patients with atrial fibrillation, a population at increased risk for both ischemic and bleeding events. This systematic review evaluates the comparative efficacy and safety of direct oral anticoagulants (DOACs) versus vitamin K antagonists (VKAs) in elderly patients with nonvalvular atrial fibrillation. A comprehensive literature search was conducted across PubMed, Embase, Scopus, and the Cochrane Central Register of Controlled Trials (CENTRAL), following the Preferred Reporting Items for Systematic Reviews and Meta-Analyses (PRISMA) guidelines, and included only English-language randomized controlled trials (RCTs) focusing on patients aged 70 years or older. Five high-quality RCTs met the eligibility criteria. The findings consistently support the non-inferiority or superiority of DOACs compared to warfarin in preventing stroke, with a significantly lower risk of intracranial hemorrhage. However, a higher incidence of extracranial bleeding, particularly gastrointestinal bleeding, was observed with certain DOACs, especially at higher doses in patients aged 80 years and older. The review also highlights a possible role of anticoagulation in cognitive protection, although current evidence is limited. Overall, DOACs appear to be an effective and generally safer alternative to VKAs in elderly patients, but individualized treatment decisions remain essential, particularly in those with advanced age, renal impairment, or elevated bleeding risk.

## Introduction and background

Atrial fibrillation (AF) is the most prevalent sustained cardiac arrhythmia globally, with its incidence rising significantly among the elderly [1]. Characterized by uncoordinated atrial activation and impaired mechanical contraction, AF is a major risk factor for cardioembolic stroke, which is associated with substantial morbidity and mortality, especially in individuals over the age of 65 [2,3]. The elderly population bears a disproportionate burden of AF-related complications due to age-related physiological changes, comorbidities, and increased vulnerability to both thromboembolic events and treatment-related adverse effects. Consequently, effective anticoagulation strategies in this demographic are critical for stroke prevention and overall risk reduction [4].

Traditionally, vitamin K antagonists (VKAs), particularly warfarin, have been the cornerstone of anticoagulation therapy in AF [5]. While VKAs have proven efficacy, they require regular international normalized ratio (INR) monitoring, are affected by numerous dietary and drug interactions, and have a narrow therapeutic window, often leading to suboptimal adherence and variable outcomes in elderly patients. The emergence of direct oral anticoagulants (DOACs), including dabigatran, rivaroxaban, apixaban, and edoxaban, has revolutionized anticoagulant therapy due to their fixed dosing, predictable pharmacokinetics, and fewer food and drug interactions [6,7]. Several large-scale randomized controlled trials (RCTs) and subsequent subgroup analyses have investigated the comparative efficacy and safety of DOACs versus VKAs in various age groups, including the elderly, with conflicting findings in terms of stroke prevention, bleeding risk, and cognitive outcomes.

Given the physiological heterogeneity of the aging population and their increased susceptibility to both ischemic events and hemorrhagic complications, it is essential to critically evaluate existing literature focusing on this high-risk group. A systematic comparison of DOACs and VKAs in elderly patients with AF can provide valuable insights for clinicians seeking to balance thromboembolic protection with bleeding risk, especially in light of updated guidelines and an expanding body of evidence.

This systematic review aims to synthesize current evidence on the comparative effectiveness and safety of DOACs versus VKAs in preventing stroke among elderly patients with AF. The review is guided by a structured population, intervention, comparator, and outcomes of interest (PICO) framework [8], which will be detailed in the eligibility criteria section.

## Review

Materials and methods

Search Strategy

The search strategy for this systematic review was designed in accordance with the Preferred Reporting Items for Systematic Reviews and Meta-Analyses (PRISMA) guidelines [9] to ensure methodological rigor and transparency. A comprehensive literature search was conducted across major medical databases including PubMed, Embase, Scopus, and the Cochrane Central Register of Controlled Trials (CENTRAL), focusing on studies published in English. The search was limited to clinical trials to ensure high-quality evidence, and filters were applied to include only RCTs evaluating the efficacy and safety of DOACs compared to VKAs in elderly patients (typically ≥70 years) with nonvalvular AF. Boolean operators and controlled vocabulary terms (e.g., Medical Subject Headings (MeSH)) were utilized to refine results, using search strings combining terms such as "atrial fibrillation", "elderly", "dabigatran", "rivaroxaban", "edoxaban", "warfarin", "stroke prevention", and "bleeding". Reference lists of included studies were also manually screened to identify additional eligible trials. The selection process, including screening, full-text review, and data extraction, was independently performed and verified to minimize bias.

Eligibility Criteria

Studies were eligible for inclusion if they were RCTs published in English and involved elderly patients, typically aged 70 years or older, with a confirmed diagnosis of nonvalvular AF. The review was guided by the PICO framework: the population included elderly patients (typically ≥70 years) with nonvalvular AF; the intervention was the administration of DOACs, such as dabigatran, rivaroxaban, or edoxaban; the comparator was treatment with a VKA, primarily warfarin; and the outcomes of interest were the incidence of ischemic or hemorrhagic stroke, systemic embolism, and major bleeding events.

Only studies that directly compared at least one DOAC with a VKA were considered eligible. Trials involving valvular AF, patients with prosthetic heart valves, pediatric populations, or studies lacking age-stratified or DOAC-specific data were excluded to maintain focus on the elderly nonvalvular AF population.

Data Extraction

Data from the included studies were extracted using a structured form by two independent reviewers to ensure accuracy and consistency. Extracted information included study design, author and year, sample size and age distribution, type and dosage of DOAC used, comparator details, duration of follow-up, and primary clinical outcomes such as stroke/systemic embolism and bleeding events. Key statistical measures such as hazard ratios (HR), confidence intervals, and p-values were also recorded to support comparative analysis. Any disagreements between reviewers were resolved through discussion or consultation with a third reviewer. The extracted data were then organized into summary tables to facilitate structured interpretation and synthesis.

Data Analysis and Synthesis

Given the clinical and methodological heterogeneity across studies, such as variations in DOAC types and dosing regimens, definitions of major bleeding, and patient age ranges, a narrative synthesis approach was adopted rather than a formal meta-analysis. The analysis focused on evaluating consistency in efficacy outcomes (i.e., stroke prevention) and safety outcomes (i.e., intracranial vs. extracranial bleeding) across the included trials. Particular attention was paid to age-dependent trends in bleeding risk and the comparative performance of DOACs versus VKAs in patients aged ≥75 or ≥80 years. The findings were discussed in light of existing literature, and the risk of bias for each study was assessed using the Cochrane Risk of Bias 2.0 (RoB 2.0) tool [10], allowing for a balanced interpretation of the strength and reliability of the evidence presented.

Results

Study Selection Process

**Figure 1:**
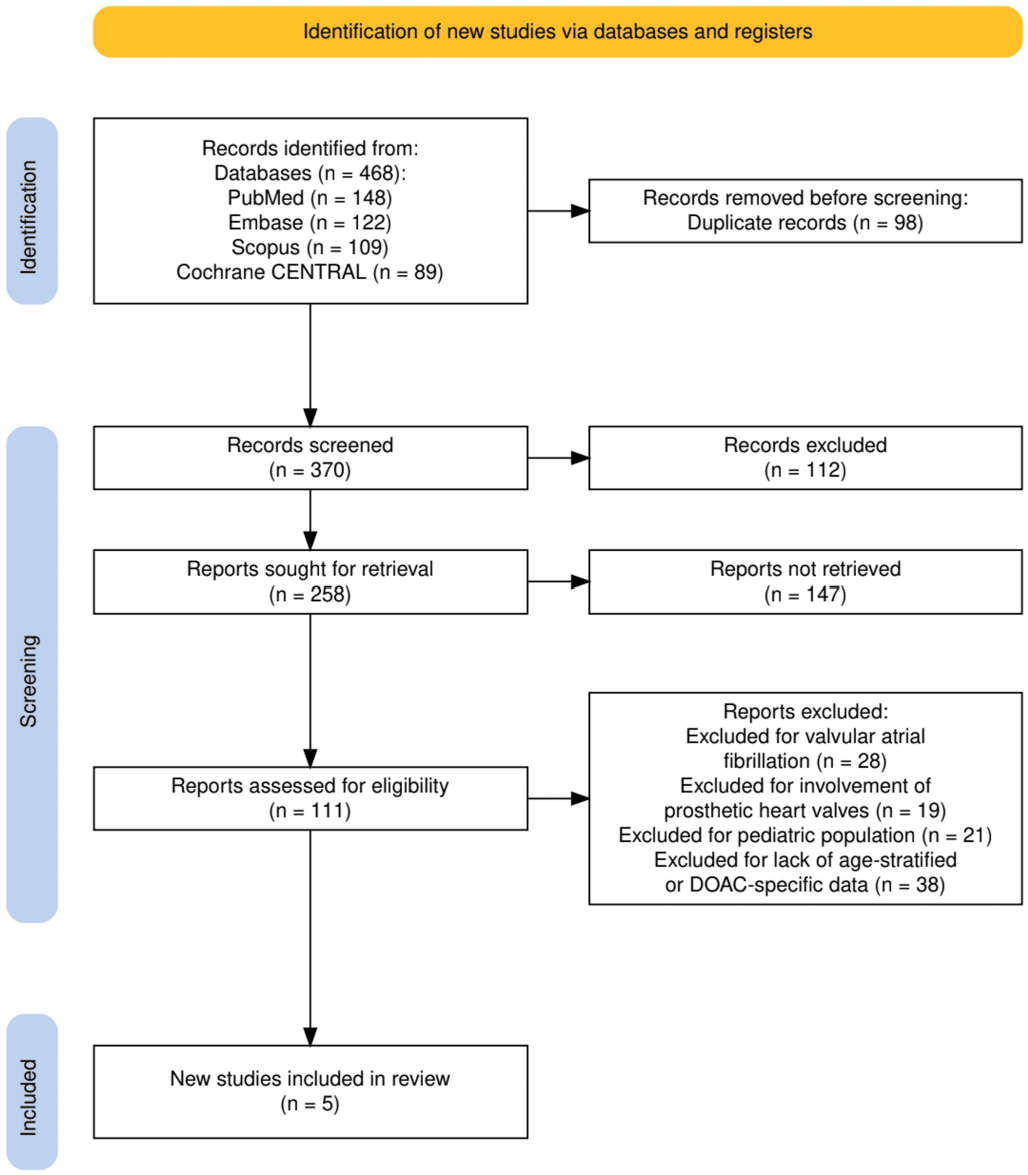
The PRISMA flowchart represents the study selection process. PRISMA: Preferred Reporting Items for Systematic Reviews and Meta-Analyses; CENTRAL: Central Register of Controlled Trials; DOAC: direct oral anticoagulants

The study selection process followed PRISMA 2020 guidelines and is illustrated in Figure 1. A total of 468 records were identified through four electronic databases: PubMed (n = 148), Embase (n = 122), Scopus (n = 109), and Cochrane CENTRAL (n = 89). After the removal of 98 duplicate records, 370 unique studies were screened based on title and abstract. Of these, 112 records were excluded for not meeting basic relevance or methodological criteria. The full texts of 258 reports were sought for further evaluation; however, 147 could not be retrieved. A total of 111 articles were assessed for eligibility, and 106 were excluded based on predefined criteria: 28 due to the involvement of valvular AF, 19 for including patients with prosthetic heart valves, 21 for focusing on pediatric populations, and 38 for lacking age-stratified or DOAC-specific data. Ultimately, five high-quality RCTs met the inclusion criteria and were included in the final qualitative synthesis.

Characteristics of the Selected Studies

The characteristics of the five studies included in this systematic review are summarized in Table 1. All selected studies were RCTs that directly compared the efficacy and safety of DOACs, including dabigatran, rivaroxaban, and edoxaban, with warfarin, a VKA, in elderly patients with nonvalvular AF. The study populations primarily consisted of individuals aged 70 years and above, with several trials specifically analyzing subgroups aged ≥75 or ≥80 years. Follow-up durations ranged from 24 months to approximately 2.8 years. While most studies reported on stroke prevention and major bleeding outcomes, one also assessed cognitive performance as a secondary endpoint. The interventions included both standard and reduced doses of DOACs, and comparators were adjusted-dose warfarin with target INR ranges of 2-3. The diversity in geographic settings, sample sizes, and outcome measures among these trials reflects a comprehensive and clinically relevant evaluation of anticoagulant therapy in an aging population.

**Table 1:** The characteristics of all the included studies. RCT: randomized controlled trial; N: sample size (number of participants); DOAC: direct oral anticoagulant; VKA: vitamin K antagonist; BID: bis in die (twice a day); INR: international normalized ratio; TTR: time in therapeutic range; MoCA: Montreal Cognitive Assessment; MMSE: Mini-Mental State Examination; NTB: Neuropsychological Test Battery; CGNT: Computerized Grooved Pegboard Neuropsychological Test; OD: omni die (once daily); CrCl: creatinine clearance; SEE: systemic embolic events; HR: hazard ratio; CI: confidence interval; Δ: delta (change); GIRAF: CoGnitive Impairment Related to Atrial Fibrillation; ENGAGE AF-TIMI 48: Effective Anticoagulation with Factor Xa Next Generation in Atrial Fibrillation-Thrombolysis in Myocardial Infarction 48; ROCKET AF: Rivaroxaban Once Daily, Oral, Direct Factor Xa Inhibition Compared With Vitamin K Antagonism for Prevention of Stroke and Embolism Trial in Atrial Fibrillation; RE-LY: Randomized Evaluation of Long-Term Anticoagulant Therapy; NS: not significant

Study (author, year)	Study design	Population (age, N)	Intervention (DOAC)	Comparator (VKA)	Follow-up duration	Primary outcome(s)	Key findings	Statistical data
Lauw et al., 2017 [11]	Post hoc analysis of RE-LY (RCT)	≥75 years; N = 18,113 (subgroup stratified by age)	Dabigatran 110 mg and 150 mg BID	Warfarin (INR 2-3)	Median 2 years	Stroke, intracranial bleeding, extracranial bleeding, mortality	Stroke and intracranial bleeding outcomes consistent across ages. Extracranial bleeding increased in ≥80 years with dabigatran, especially 150 mg BID	HR for stroke (150 mg): 0.63-0.70; HR for extracranial bleeding ≥80 years: 1.68 (150 mg), 1.50 (110 mg); interaction p < 0.001
Caramelli et al., 2022 (GIRAF) [12]	RCT (open-label, parallel-group)	≥70 years; N = 200	Dabigatran 110/150 mg BID	Warfarin (TTR ~70%)	24 months	Cognitive decline (MoCA, MMSE, NTB, CGNT)	No significant difference in cognitive decline between groups; MoCA slightly favored warfarin but not clinically meaningful	MoCA Δ = -0.96 (95% CI: -1.80 to 0.13; p = 0.02); MMSE Δ = -0.12 (p = 0.75); all adjusted p > 0.05
Yamashita et al., 2016 (ENGAGE AF-TIMI 48) [13]	Prespecified RCT subgroup analysis	≥21 years (East Asians); N = 1,943	Edoxaban 60 mg and 30 mg OD	Warfarin (INR 2-3)	~2.8 years	Stroke/systemic embolism, major bleeding	High-dose edoxaban superior to warfarin for stroke prevention and major bleeding; low-dose non-inferior for stroke with lowest bleeding risk	Stroke/SEE: HR 0.53 (60 mg; p = 0.02); major bleeding: HR 0.61 (60 mg), 0.34 (30 mg); p < 0.001
Halperin et al., 2014 (ROCKET AF) [14]	RCT, prespecified secondary analysis	≥75 years; N = 6,229	Rivaroxaban 20 mg OD (15 mg if CrCl <50)	Warfarin (INR 2-3)	~2 years	Stroke/systemic embolism, major bleeding	Rivaroxaban non-inferior to warfarin in elderly patients; consistent efficacy and safety across age groups	Stroke/SEE ≥75 years: HR 0.80 (95% CI: 0.63-1.02); major bleeding: HR 1.11 (95% CI: 0.92-1.34); interaction p = NS
Eikelboom et al., 2011 (RE-LY) [15]	RCT (bleeding-focused subgroup analysis)	≥75 years vs. <75 years; N = 18,113	Dabigatran 110 and 150 mg BID	Warfarin (INR 2-3)	Median 2 years	Major bleeding (intracranial and extracranial)	Dabigatran safer in <75 years; in ≥75, lower intracranial bleeding but higher or similar extracranial bleeding with both doses	≥75 years: dabigatran 110 mg vs. warfarin: 4.43% vs. 4.37% (p = 0.89); dabigatran 150 mg: 5.10% vs. 4.37% (p = 0.07); interaction p < 0.001

Quality Assessment

The quality assessment of the included studies, as presented in Table 2, was conducted using the Cochrane RoB 2.0 tool, which evaluates several domains including randomization, adherence to intended interventions, completeness of outcome data, accuracy of outcome measurement, and selective reporting. Overall, three of the five studies were rated as having a low risk of bias across all domains, reflecting robust methodological quality and reliable outcome reporting. Two studies were assessed as having some concerns, one due to its open-label design, which may introduce performance or detection bias, and the other because it involved post hoc subgroup analysis, which carries inherent limitations in statistical power and reporting clarity. Nonetheless, all studies were deemed of sufficient quality to contribute meaningful evidence to the review, with none classified as having a high risk of bias. This consistent methodological rigor across trials strengthens the validity of the synthesized findings.

**Table 2:** The overall risk of bias assessment of all the included studies. RoB 2.0: Risk of Bias 2.0; GIRAF: CoGnitive Impairment Related to Atrial Fibrillation; ENGAGE AF-TIMI 48: Effective Anticoagulation with Factor Xa Next Generation in Atrial Fibrillation-Thrombolysis in Myocardial Infarction 48; ROCKET AF: Rivaroxaban Once Daily, Oral, Direct Factor Xa Inhibition Compared With Vitamin K Antagonism for Prevention of Stroke and Embolism Trial in Atrial Fibrillation; RE-LY: Randomized Evaluation of Long-Term Anticoagulant Therapy

Study (author, year)	Tool used	Randomization process	Deviations from intended interventions	Missing outcome data	Measurement of the outcome	Selection of the reported result	Overall risk of bias
Lauw et al., 2017 [11]	RoB 2.0	Low	Low	Low	Low	Low	Low
Caramelli et al., 2022 (GIRAF) [12]	RoB 2.0	Low	Some concerns (open-label design)	Low	Low	Low	Some concerns
Yamashita et al., 2016 (ENGAGE AF-TIMI 48) [13]	RoB 2.0	Low	Low	Low	Low	Low	Low
Halperin et al., 2014 (ROCKET AF) [14]	RoB 2.0	Low	Low	Low	Low	Low	Low
Eikelboom et al., 2011 (RE-LY) [15]	RoB 2.0	Low	Low	Low	Low	Some concerns (subgroup post hoc focus)	Some concerns

Discussion

The findings of this systematic review highlight the comparative effectiveness and safety of DOACs versus VKAs in elderly patients with AF. Across all five included RCTs, DOACs demonstrated comparable or superior efficacy in preventing stroke and systemic embolism when compared to warfarin, with generally consistent performance across age groups. In the Randomized Evaluation of Long-Term Anticoagulant Therapy (RE-LY) subgroup analysis by Lauw et al. [11], dabigatran at 150 mg bis in die (twice a day) (BID) showed strong stroke prevention with an HR ranging from 0.63 to 0.70 compared to warfarin. However, the risk of extracranial bleeding significantly increased in patients aged ≥80 years, particularly with the 150 mg dose (HR: 1.68) and to a lesser extent with the 110 mg dose (HR: 1.50), with a notable interaction (p < 0.001). Similarly, the Rivaroxaban Once Daily, Oral, Direct Factor Xa Inhibition Compared With Vitamin K Antagonism for Prevention of Stroke and Embolism Trial in Atrial Fibrillation (ROCKET AF) [14] secondary analysis by Halperin et al. reported that rivaroxaban was non-inferior to warfarin for stroke/systemic embolism prevention in patients aged ≥75 years (HR: 0.80; 95% CI: 0.63-1.02), while bleeding risks were comparable (HR: 1.11; 95% CI: 0.92-1.34). The Effective Anticoagulation with Factor Xa Next Generation in Atrial Fibrillation-Thrombolysis in Myocardial Infarction 48 (ENGAGE AF-TIMI 48) East Asian subgroup [13] showed that high-dose edoxaban significantly reduced both stroke risk (HR: 0.53; p = 0.02) and major bleeding (HR: 0.61; p = 0.011) compared to warfarin, while the low-dose regimen had the lowest bleeding rate (HR: 0.34; p < 0.001) with non-inferior efficacy. Eikelboom et al. [15] confirmed that although dabigatran lowered intracranial bleeding at all ages, extracranial bleeding was more frequent in those aged ≥75 years, particularly with the 150 mg dose (5.10% vs. 4.37%; p = 0.07), with a significant age-treatment interaction (p < 0.001). Meanwhile, the CoGnitive Impairment Related to Atrial Fibrillation (GIRAF) trial [12] focusing on cognitive outcomes found no statistically significant differences between dabigatran and warfarin users in terms of cognitive decline over two years, with adjusted p-values exceeding 0.05 for all primary cognitive domains. Overall, these findings support the efficacy of DOACs as alternatives to VKAs in elderly patients but underscore the importance of individualized risk assessment, especially concerning bleeding risk in those over 75 or 80 years of age.

The findings of this review align closely with existing literature and current guideline recommendations, reinforcing the preferential use of DOACs over VKAs in elderly patients with nonvalvular AF. Major clinical guidelines, including those from the European Society of Cardiology (ESC) [16] and the American Heart Association/American College of Cardiology/Heart Rhythm Society (AHA/ACC/HRS), advocate for DOACs as first-line agents due to their favorable safety profiles, predictable pharmacokinetics, and reduced need for monitoring [17]. Our review supports these recommendations by demonstrating that DOACs provide comparable, if not superior, stroke prevention while significantly reducing the risk of intracranial hemorrhage, a particularly devastating complication in elderly patients. These findings are consistent with prior meta-analyses, such as those by Ruff et al. [18] and López-López et al. [19], which have shown lower rates of hemorrhagic stroke and fatal bleeding with DOACs compared to warfarin. However, our review also underscores concerns specific to the elderly population, particularly the increased risk of extracranial bleeding, including gastrointestinal hemorrhage, in patients aged ≥75 or ≥80 years, especially when using higher doses of dabigatran. These age-related bleeding patterns highlight the importance of individualized dosing strategies and close clinical monitoring in geriatric populations [20]. Overall, our analysis affirms the broader literature while drawing attention to nuanced risk-benefit considerations that are especially pertinent in the management of anticoagulation among older adults.

The findings of this review have important clinical implications for the management of elderly patients with AF, particularly in selecting the most appropriate anticoagulant regimen. DOACs consistently demonstrated comparable or superior efficacy to VKAs in stroke prevention and were associated with a significantly lower risk of intracranial hemorrhage. However, in patients aged ≥80 years, caution is warranted due to a heightened risk of extracranial bleeding, especially with higher doses of dabigatran [21]. These results underscore the need for individualized treatment strategies that consider age, renal function, and bleeding risk. For example, using 110 mg of dabigatran in very elderly or renally impaired individuals may help balance efficacy and safety [22]. The potential role of DOACs in cognitive protection, though not yet definitive, is an emerging area that may influence prescribing patterns in the future [23]. These insights are particularly relevant for clinicians involved in long-term anticoagulation management, including primary care physicians, geriatric cardiologists, and stroke specialists.

This review offers several strengths: it includes high-quality RCTs, many of which feature prespecified subgroup analyses focusing on elderly patients, a group often underrepresented in clinical research. The methodology adheres closely to PRISMA guidelines, ensuring transparency and reproducibility. By narrowing the focus to this high-risk demographic, the review provides clinically meaningful, age-specific insights.

Nonetheless, certain limitations should be noted. The review includes only five studies, which may limit generalizability across different populations. Some studies were based on subgroup or post hoc analyses, potentially introducing bias. Cognitive outcomes were assessed in only one trial (the GIRAF study), and its open-label design may affect the reliability of those findings [12].

Methodological variability among the included studies, such as differing definitions of major bleeding, variations in DOAC dosing, and follow-up durations, may also contribute to heterogeneity. Additionally, long-term outcomes like dementia progression, functional decline, and quality of life were underexplored. Notably, data remain sparse for frail elderly individuals, those over 85 years, and patients with multimorbidity or polypharmacy, who represent a substantial portion of real-world AF cases.

Future research should prioritize large, prospective trials focused on patients aged ≥80 or ≥85 years, incorporating real-world factors like medication adherence, polypharmacy, and functional status to improve external validity. Head-to-head comparisons between DOACs, such as apixaban versus rivaroxaban, are also needed to guide optimal drug selection in elderly patients [24]. Furthermore, additional studies should investigate the long-term cognitive effects of anticoagulant therapy, as there is growing interest in its potential role in preserving cognitive function in AF [25].

## Conclusions

This systematic review reinforces the clinical value of DOACs as effective and generally safer alternatives to VKAs for stroke prevention in elderly patients with AF. The evidence demonstrates that while DOACs offer comparable or superior efficacy in reducing thromboembolic events and significantly lower the risk of intracranial hemorrhage, their use in very elderly patients (≥80 years) warrants careful consideration due to an increased risk of extracranial bleeding, particularly at higher doses. These findings highlight the need for individualized anticoagulation strategies that take into account patient age, renal function, and bleeding risk. This review adds valuable, age-specific insights to the growing body of literature and supports guideline-directed care in geriatrics and primary care settings. Additionally, the integration of shared decision-making tools or patient decision aids may enhance treatment adherence and ensure personalized, informed choices for elderly patients facing anticoagulation therapy.

## References

[1] Salih M, Abdel-Hafez O, Ibrahim R, Nair R (2021). Atrial fibrillation in the elderly population: challenges and management considerations. J Arrhythm.

[2] Sanders GD, Lowenstern A, Borre E (2018). Stroke Prevention in Patients With Atrial Fibrillation: A Systematic Review Update. https://www.ncbi.nlm.nih.gov/books/NBK534141/.

[3] Nesheiwat Z, Goyal A, Jagtap M (2023). Atrial fibrillation. StatPearls.

[4] Parrini I, Lucà F, Rao CM (2025). Management of atrial fibrillation in elderly patients: a whole new ballgame?. J Clin Med.

[5] Zirlik A, Bode C (2017). Vitamin K antagonists: relative strengths and weaknesses vs. direct oral anticoagulants for stroke prevention in patients with atrial fibrillation. J Thromb Thrombolysis.

[6] Almarshad F, Alaklabi A, Bakhsh E, Pathan A, Almegren M (2018). Use of direct oral anticoagulants in daily practice. Am J Blood Res.

[7] Gunawardena T (2021). Direct oral anticoagulants: a review for the non-specialist. Hematol Rep.

[8] Eriksen MB, Frandsen TF (2018). The impact of patient, intervention, comparison, outcome (PICO) as a search strategy tool on literature search quality: a systematic review. J Med Libr Assoc.

[9] Page MJ, McKenzie JE, Bossuyt PM (2021). The PRISMA 2020 statement: an updated guideline for reporting systematic reviews. BMJ.

[10] Sterne JA, Savović J, Page MJ (2019). RoB 2: a revised tool for assessing risk of bias in randomised trials. BMJ.

[11] Lauw MN, Eikelboom JW, Coppens M (2017). Effects of dabigatran according to age in atrial fibrillation. Heart.

[12] Caramelli B, Yu PC, Cardozo FA (2022). Effects of dabigatran versus warfarin on 2-year cognitive outcomes in old patients with atrial fibrillation: results from the GIRAF randomized clinical trial. BMC Med.

[13] Yamashita T, Koretsune Y, Yang Y (2016). Edoxaban vs. warfarin in East Asian patients with atrial fibrillation - an ENGAGE AF-TIMI 48 subanalysis. Circ J.

[14] Halperin JL, Hankey GJ, Wojdyla DM (2014). Efficacy and safety of rivaroxaban compared with warfarin among elderly patients with nonvalvular atrial fibrillation in the Rivaroxaban Once Daily, Oral, Direct Factor Xa Inhibition Compared With Vitamin K Antagonism for Prevention of Stroke and Embolism Trial in Atrial Fibrillation (ROCKET AF). Circulation.

[15] Eikelboom JW, Wallentin L, Connolly SJ (2011). Risk of bleeding with 2 doses of dabigatran compared with warfarin in older and younger patients with atrial fibrillation: an analysis of the randomized evaluation of long-term anticoagulant therapy (RE-LY) trial. Circulation.

[16] Bhandari M, Pradhan A, Vishwakarma P, Di Renzo L, Iellamo F, Ali W, Perrone MA (2025). Direct oral anticoagulant use in older adults with atrial fibrillation: challenges and solutions. Eur Cardiol.

[17] Joglar JA, Chung MK, Armbruster AL (2024). 2023 ACC/AHA/ACCP/HRS guideline for the diagnosis and management of atrial fibrillation: a report of the American College of Cardiology/American Heart Association Joint Committee on Clinical Practice Guidelines. J Am Coll Cardiol.

[18] Ruff CT, Giugliano RP, Braunwald E (2014). Comparison of the efficacy and safety of new oral anticoagulants with warfarin in patients with atrial fibrillation: a meta-analysis of randomised trials. Lancet.

[19] López-López JA, Sterne JA, Thom HH (2017). Oral anticoagulants for prevention of stroke in atrial fibrillation: systematic review, network meta-analysis, and cost effectiveness analysis. BMJ.

[20] Zhou Z, Slattum PW, Ke A, Zhang L (2023). Managing drug-drug interactions in older adults. J Clin Pharmacol.

[21] Avgil-Tsadok M, Jackevicius CA, Essebag V, Eisenberg MJ, Rahme E, Behlouli H, Pilote L (2016). Dabigatran use in elderly patients with atrial fibrillation. Thromb Haemost.

[22] Chen A, Stecker E, Warden B (2020). Direct oral anticoagulant use: a practical guide to common clinical challenges. J Am Heart Assoc.

[23] Lucà F, Oliva F, Abrignani MG (2023). Management of patients treated with direct oral anticoagulants in clinical practice and challenging scenarios. J Clin Med.

[24] Cohen M, Spyropoulos AC, Goodman SG (2024). Rivaroxaban versus apixaban: a comparison without a simple solution. Mayo Clin Proc Innov Qual Outcomes.

[25] Jacobs V, May HT, Bair TL (2016). Long-term population-based cerebral ischemic event and cognitive outcomes of direct oral anticoagulants compared with warfarin among long-term anticoagulated patients for atrial fibrillation. Am J Cardiol.

